# Recyclable Nanostructured Catalytic Systems in Modern Environmentally Friendly Organic Synthesis

**DOI:** 10.3390/molecules15074792

**Published:** 2010-07-08

**Authors:** Irina Beletskaya, Vladimir Tyurin

**Affiliations:** 1 A.N. Frumkin Institute of Physical Chemistry and Electrochemistry, 31, Leninskiy prosp., Moscow, 119991, Russia; E-Mail: tv@org.chem.msu.ru (I.B.); 2 Chemistry Department, M.V. Lomonosov Moscow State University, 1-3, Leninskye Gory, Moscow, 119992, Russia

**Keywords:** nanoparticles, catalysis, nanocatalysts, catalyst recycling, polymer supported catalysts, Green Chemistry

## Abstract

Modern chemical synthesis makes heavy use of different types of catalytic systems: homogeneous, heterogeneous and nano-sized. The latter – nano-sized catalysts – have given rise in the 21st century to a rapidly developing area of research encompassing several prospects and opportunities for new technologies. Catalytic reactions ensure high regio- and stereoselectivity of chemical transformations, as well as better yields and milder reaction conditions. In recent years several novel catalytic systems were developed for selective formation of carbon-heteroatom and carbon-carbon bonds. This review presents the achievements of our team in our studies on various types of catalysts containing metal nanoparticles: palladium-containing diblock copolymer micelles; soluble palladium-containing polymers; metallides on a support; polymeric metal salts and oxides; and, in addition, metal-free organic catalysts based on soluble polymers acting as nanoreactors. Representative examples are given and discussed in light of possible applications to solve important problems in modern organic synthesis.

## 1. Introduction

The fact that the size of a catalytic particle has a strong influence on its activity in various heterogeneous and heterogenized catalysis processes has been well known for a long time and this feature is widely used by chemists. However, more recently research has been focused on the creation of nano-sized structures, their stabilization, on the study of their structure and its influence on activity. Such research became possible after electronic microscopy became a customary tool, with the advent of methods like scanning electron microscopy (SЕМ), transmission electron microscopy (TEM), *etc*. As it turned out, and should have been expected, the creation of catalytic systems of well defined structure and small size not only allows one to greatly increase the catalytic activity of heterogeneous catalysts, approaching the performance levels of homogeneous ones, but also allows one to solve a range of problems directed at the creation of more economic and environmentally harmless processes in accordance with the principles of Green Chemistry. We, in particular, have investigated soluble polymers capable of self-organization in solution to form nano-sized microheterogeneous structures, showing properties of nanoreactors where the reagents concentrate and catalytic particles are held. Researchers have long been interested in self-organizing polymers and their catalytic properties, modeling the natural enzymatic systems, however, advances in supramolecular chemistry which increased the studies of intermolecular interactions and self-organization processes on a qualitatively new level, allowed the development of a systematic approach and the understanding which have led to an explosive development of research on nanostructured systems. A major part of our work is directed at the creation of the recyclable catalytic systems for the cross-coupling reactions based on palladium nanoparticles (PdNPs). The main problem of such systems is related to leaching of the palladium which limits the reuse of the catalyst. On the other hand many reactions being homogeneously catalyzed processes proceed because of leaching. This is the usual case in the catalysis by metal nanoparticles although there are a few truly heterogeneous reactions proceeding on the surface of nanoparticles and the reactions catalyzed by metal salts are among them. Even if the leaching problem is solved, the nanoparticles cannot be used infinitely as the size and the morphology of the particles are changing during the course of the reaction and eventually, the growth of the particles leads to a loss of activity. The situation is different in the case of organic catalysis. There can be no change in the structure of the organic catalyst and no leaching of the active metal, and if the reaction products don’t poison the catalyst, it should work infinitely. The absence of transition metals is an additional advantage which is important in the synthesis of pharmaceutical products. Born in the last decade organocatalysis currently seems to be in a “gold rush” state. 

In this work we have reviewed various types of metal-containing nanocatalysts: colloidal palladium stabilized by surfactants in water, palladium-containing diblock copolymer micelles; soluble palladium-containing polymers; metallides on a support; polymeric metal salts and oxides; and, besides, organic catalysts not containing metal but rather consisting of enzyme-like soluble polymers acting as nanoreactors. Representative examples are given and discussed with a view to solving important problems in modern organic synthesis.

## 2. Palladium Nanoparticles

Metal nanoparticles as representatives of the most elementary class of nanoobjects – nanopowders – were first obtained a rather long time ago. A catalyst such as a palladium on charcoal, representing the PdNPs fixed on activated charcoal, has been known for many years and is widely used both in industry, and in laboratories, but as a heterogeneous catalyst. PdNPs are often formed during homogeneous catalytic reactions as a consequence of the reduction of Pd(II) to the native metal form [1,2,3]. Over the last two decades interest in metal nanoparticles has increased considerably. The nanoparticles stabilized by some sort of support could have several important advantages over both traditional homogeneous and heterogeneous catalysts. The major drawbacks of homogeneous catalysts are their difficult separation from the reaction products and the impossibility of reuse. Heterogeneous catalysts don’t have these problems, but such catalysts are less active and gradually lose their activity because of leaching. Nanoparticles relate to the microheterogeneous catalysts which combine the high activity of homogeneous catalysts and the ease of separation and reuse of heterogeneous catalysts. Following the tradition, we will refer to the catalyst and also its precursors (pre-catalysts), although the forms really participating in a catalytic cycle can represent single metal atoms together with the ligand environment, "pulled out" of a metal nanoparticle by reagents or formed from other initial compounds of this metal. In most cases, metal leaching can be responsible for the reaction which proceeds homogeneously, even if the catalyst is heterogeneous or microheterogeneous. At the end of a reaction the metal atoms again form a nanoparticle, and the morphology of particles and their size can change. Fine particles often aggregate to form inactive larger particles. For successful use in catalysis and furthermore for repeated use, it is necessary to stabilize the nanoparticles, to isolate them from each other and thus to prevent aggregation. This is achieved by application of surfactants (in emulsions), by use of ionic liquids, and by using metals dispersed on organic and inorganic heterogeneous supports (clay, activated charcoal, chitosan, *etc.*), or on synthetic polymers [4]. Fastening of nanoparticles on supports, easily separated from reaction products, allows multiple use of the catalyst. However, there are some problems: irreversible leaching of metal from the support and Ostwald ripening of the nanoparticles, leading to loss of activity. Some researchers claim that some cross-coupling reactions can proceed on the surface of metal nanoparticle [5,6] but in most cases leaching of metal from the nanoparticle to solution is a necessary reaction step [7]. Therefore the support should catch atoms or nanoparticles formed from atoms left by the catalytic cycle in the solution. On the other hand the support should maintain the size and morphology of the nanoparticles. One of the ways to solve this problem is to use nanostructured materials which favor particles of a specific form. Metal - support interactions are very important for the catalytic activity and selectivity. The deformation of the nanoparticles adsorbed to the surface leads to the change of the energy of different atoms in the catalytic clusters and results in the modification of their reactivity [7]. Mass transport is another problem of supported catalysts manifesting in that an increase in stability often leads to a decrease in activity. Many efforts of researchers have concentrated on finding supports which have the optimal balance between strong holding of metal nanoparticles and their accessibility to reagents. 

The basic purposes which we pursued in creating nanosized palladium catalysts, can be formulated as follows: 1) the catalyst should not contain toxic phosphine ligands; 2) the catalyst can be used in water and water-organic mixtures, *i.e.,* the cheapest and green solvents; 3) and, finally, the catalyst should be easily separable from a product and, whenever possible, should retain its catalytic activity. Such conditions completely satisfy Green Chemistry principles [8].

### 2.1. Ligandless palladium in water. Reactions in microemulsions

It should be noted that the catalysis of cross-coupling reactions with a zero-valent palladium species in water was carried out by us for the first time in 80s [9,10]. The reaction was performed without addition of phosphine ligands and, according to modern representations, the true catalyst (or pre-catalyst) was constituted by PdNPs, probably stabilized by adsorbed anions. Since the aggregation of metal in the absence of stabilizing ligand could be prevented by a fast reaction of a reagent with Pd(0), such systems could only be used with very active aryl halides. Therefore in case of less active substrates researchers have been compelled to use water-soluble phosphine complexes of palladium, *i.e.,* to realize reactions under usual homogeneous conditions with participation of metallocomplexes [11,12].

**Scheme 1:**
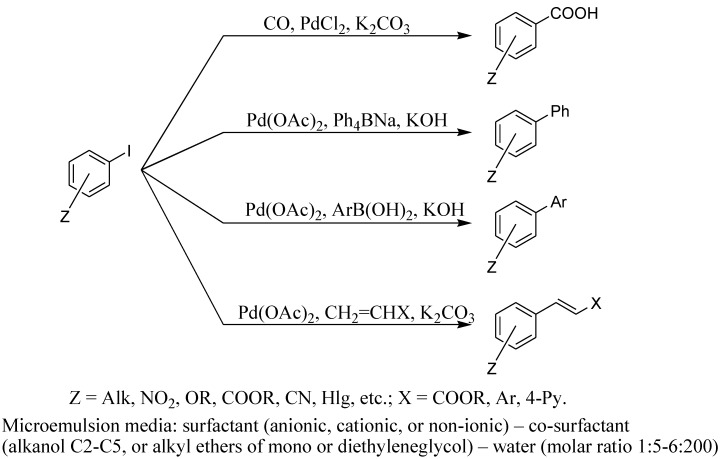
Palladium catalyzed reactions in microemulsion.

**Figure 1:**
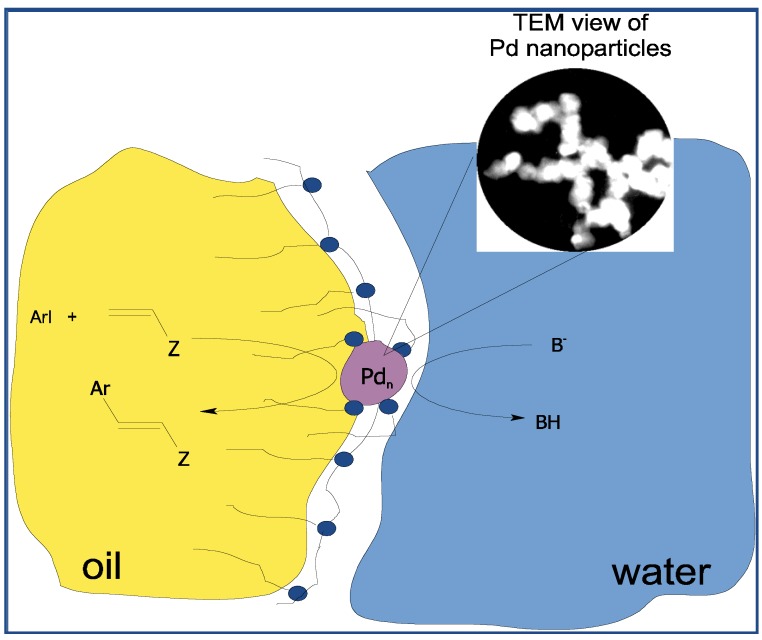
PdNPs in a microemulsion.

We suggested the use of microemulsions consisting of various types of surfactants, for example, C_17_H_33_COOK and butanol as co-surfactant, as stabilizing systems for PdNPs. The reduction of PdX_2_ during cross-coupling reactions led to formation of palladium metal colloid with PdNPs of an average size of 10 nm stabilized by the action of surfactants. It is interesting to note that the catalyst could be stored in such state for a long time and can be used repeatedly [13]. A number of catalytic reactions [the Suzuki-Miyaura reaction, arylation of olefins (the Heck reaction) and carbonylation of aryl iodides] were successfully carried out with this technique (Scheme 1). The reactions proceeded in the system of drop of oil in water (Figure 1). The yield of the reaction correlates with the stability of the Pd sol (Table 1). 

**Table 1:** The correlation of performance of microemulsion media (surfactant - n-butanol - water) in the Heck arylation with the stability of palladium sol in the same system.

Surfactant	Catalyst (mol %)	Time, h	Yield, %	Sol stability ^1^
C_17_H_33_COOK	PdCl_2_ (0.01)	8	90	+++
C_15_H_31_COOK	PdCl_2_ (0.01)	8	78	+++
C_13_H_27_COOK	PdCl_2_ (0.1)	8	32	++
C_11_H_23_COOK	PdCl_2_ (0.1)	8	10	+
C_16_H_33_NMe_3_Br	PdCl_2_ (0.01)	8	86	+++
C_16_H_33_SO_3_K	PdCl_2_ (0.1)	8	4	-
C_17_H_33_COOK	Pd^2^ (0.1)	3	40	+++
C_17_H_33_COOK	Pd^3^ (0.1)	3	47	+++
C_17_H_33_COOK	Pd^4^ (0.1)	3	39	+++

^1^ Qualitative measure of sol stability in the reaction mixture. +++ the sol rapidly forms and shows no signs of degradation during the reaction; ++ the sol is formed readily, but shows partial aggregation and sedimentation; + the sol is formed but readily undergoes sedimentation; - the sol is not formed at all; ^2 ^Palladium sol freshly prepared in the same microemulsion; ^3^ Same sol aged for 3 months; ^4^ Same sol aged for 1 year.

The obvious drawbacks of this system are the difficulties of separation of the colloidal catalyst and the necessity to use only aryl iodides, the same as in the corresponding reaction in water without ligands.

### 2.2. Palladium nanoparticles stabilized by water-soluble diblock copolymeric micelles

Polymer supports have become commonly used in catalysis. Polymers effectively stabilize nanoparticles and are also used as phase anchors for catalysts to facilitate their recovery, separation, and reuse. Soluble polymers are among the very promising supports for catalysts because of the absence of mass transport problems. Soluble polymer-bound analogs of low molecular weight catalysts often have the same activity as their low molecular weight counterparts [14]. Amphiphilic diblock copolymers are particular of interest for the immobilization of nanoparticles [15,16].

**Figure 2:**
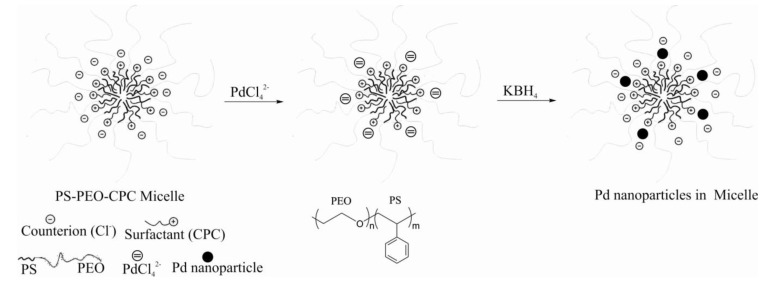
The scheme of formation of micellar system.

Diblock copolymers consisting of lyophilic and lyophobic blocks, upon dissolution in a selective solvent (dissolving only one block), self-organize in micelles in which the lyophobic block forms a dense core, and lyophilic block forms a diffuse micelle corona. In water the hydrophobic block forms a core, and the hydrophilic one, a corona of a micelle. On the contrary, in a nonpolar solvent the core contains the hydrophilic block, therefore the metal nanoparticles immobilized due to interaction with the hydrophilic block are located in the core of the micelle. The latter case is represented by PdNPs supported by polystyrene-polyvinylpyridine diblock copolymer in toluene [17]. The activity of such catalysts was low because of inaccessibility of the PdNPs inside the core to reagents. PdNPs immobilized on diblock copolymer polystyrene-poly(sodium acrylate) in water are located in the diffuse corona of the micelle and are easily accessible to reagents. But in this case the friable corona holds the PdNPs weakly [18]. Therefore we have investigated the system essentially differing from foregoing, in which metal nanoparticles are neither in a core and nor in a corona, but rather in their interface. The amphiphilic diblock copolymer of polystyrene-poly(ethylene oxide) (PS-PEO) has been dissolved in water with addition of surfactant - a cetylpyridinium chloride (CPC), its hydrocarbon chains penetrating the hydrophobic core of a micelle formed by the polymer, with the charged groups left on the interface, giving to it a positive charge. Addition of K_2_PdCl_4_ leads to adsorption of anions of PdCl_4_^2-^ on the interface of the core of a micelle. The palladium (II) is reduced by KBH_4_, forming nanoparticles adsorbed on the interface of the core of a micelle (Figure 2). 

Thus, this catalytic system essentially differs from foregoing, because metal nanoparticles in it are accessible to reagents, and at the same time, being in the dense structured layer, they are strongly held in a micelle due to adsorption forces. This catalyst has been investigated in carbon-carbon bond formation reactions (cross-coupling reactions) [19], and has shown high activity and exclusive stability in aqueous solutions at temperatures below 60 ºC. The catalyst is easily separated with the help of nanoporous membranes from low-molecular reaction products and can be reused [20]. However, above 60 ºC de-structuring of the micelle system occurred. 

#### 2.2.1. Catalysis of heterocyclization reactions in organic solvent

**Scheme 2:**
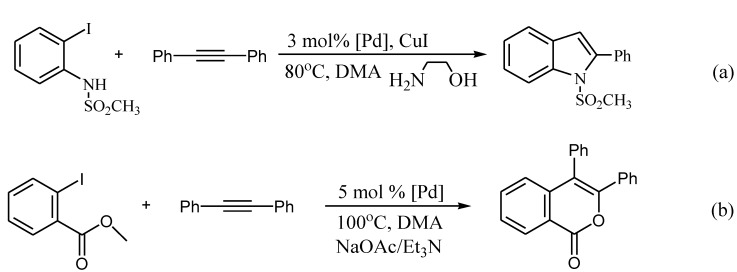
Heterocyclization reactions.

Our research on PdNPs immobilized in a diblock copolymer PS-PEO micelle has shown that this catalyst possesses high catalytic activity in the olefin arylation reaction (the Heck reaction), and, moreover, in other reactions. The latter reactions allow one to carry out with ease the synthesis of complex heterocyclic systems, such as indoles and isocoumarins [19] (Scheme 2).

**Table 2:** Fresh start of the heterocyclization reactions.

Reaction	Cycle Number	Catalyst (mol% Pd)	Time, hrs	Yield, %
a	1	3	4,5	79
a	2	3	4,5	84
a	3	3	4,5	73
b	1	0.5	10	68
b	2	0.5	10	71
b	3	0.5	10	74

However, a rather high temperature is required for these specified reactions, at which the destruction of the micelle is observed that makes impossible to separate and recycle the catalyst. Nevertheless, addition of a new portion of reagents (fresh start) after the end of the reaction allowed us to carry out several cycles without a loss of the catalyst activity (Table 2). Presumably, palladium leaching into solution is not accompanied by appreciable aggregation of the PdNPs. We shall note, that activity of the catalytic system is quite comparable with that of the homogeneous system PdCl_2_(PPh_3_)_2_, and its main advantage is the possibility of reusing it repeatedly.

#### 2.2.2. Catalysis of carbon-carbon bond formation reactions in water

**Scheme 3:**
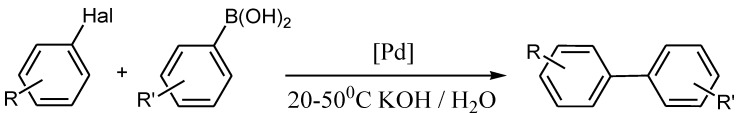
The Suzuki-Miyaura reaction.

In order to realize recycling of the catalyst, the proposed catalytic system has been studied in reaction with water-soluble reagents that allows one to carry out reactions in neat water at low temperatures (below 60 ºC). The PdNPs micellar catalyst was thus investigated in the Suzuki-Miyaura reaction in water at 20–50 ºC [20] (Scheme 3).

**Figure 3:**
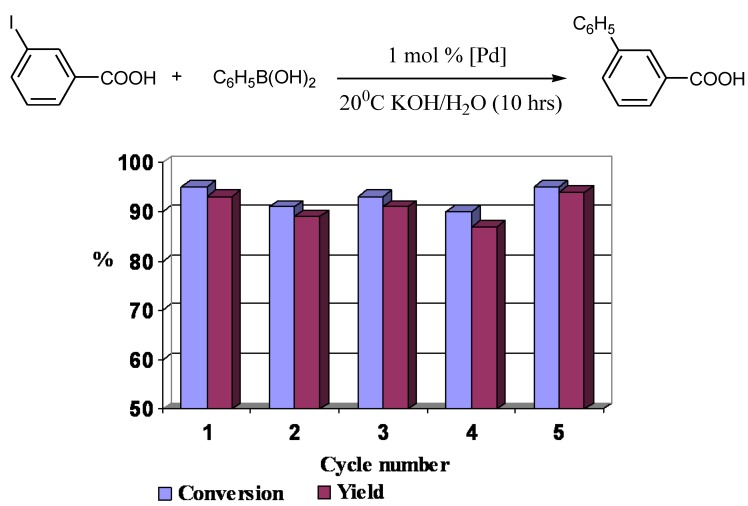
Recycling the catalyst in water after an ultrafiltration step.

In the reactions of water-soluble aromatic (heteroaromatic) iodides and activated bromides with arylboronic acids in water biaryls were formed in practically quantitative yields. The catalyst isolated after the reaction of *m*-iodobenzoic acid with phenylboronic acid by means of ultrafiltration retained constant activity for at least five cycles (Figure 3).

**Figure 4:**
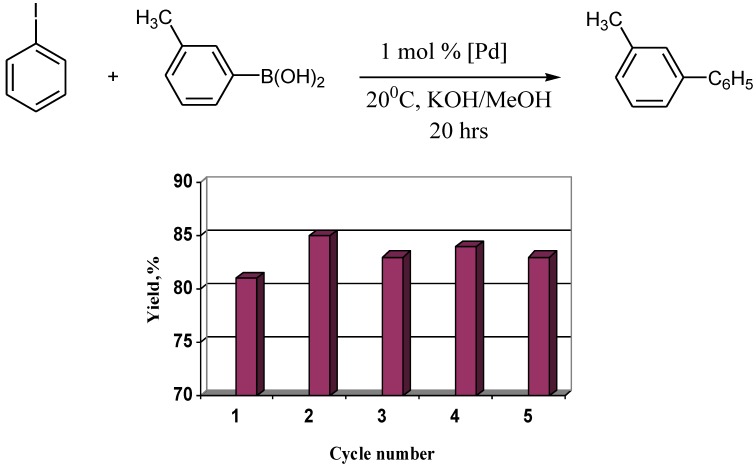
Recycling the catalyst in methanol with use of centrifugation.

Reactions with poorly soluble aryl iodides proceed with lower yields, giving arylboronic acid homocoupling by-products, consequently reactions involving water insoluble reagents were carried out in methanol. Reactions in less polar methanol required longer times and higher temperatures, up to 50 ºC. Recycling of the catalyst was carried out by ultrafiltration. Addition of a new portion of reagents to the mother liquid after the ultrafiltration led to the reaction proceeding albeit with low yield. This fact means that a small part of palladium remained in solution after the isolation of the catalyst. The best way to recycle the catalyst in methanol was found to be centrifugation (Figure 4). 

**Figure 5:**
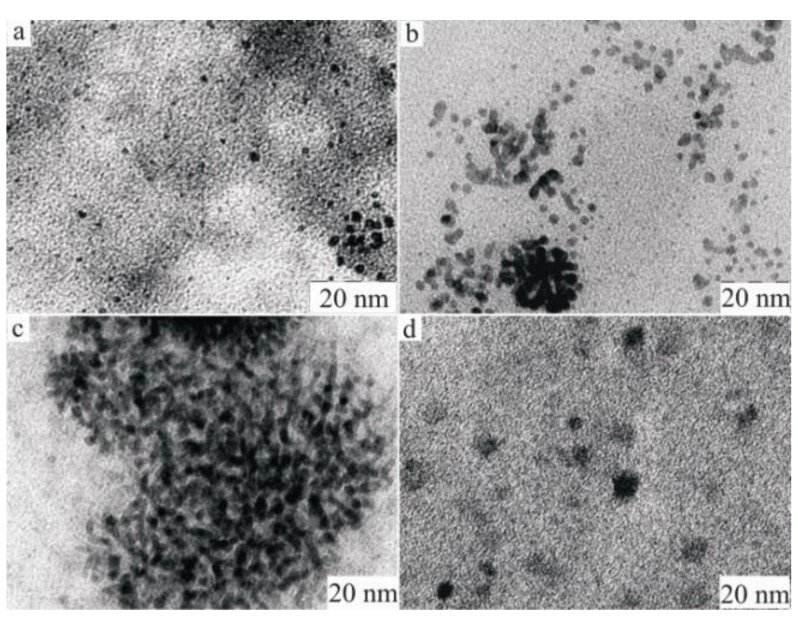
TEM images of the PdNPs before **(a)** and after (**(b)** for a liquid phase; **(c) **for the precipitated phase) the Suzuki-Miyaura reactions and after interaction of the catalyst with *m-*iodobenzoic acid at the presence of KOH in water **(d)**.

Our attempt to use centrifugation instead of ultrafiltration that could essentially reduce the time of the catalyst separation, turned out to be unsuccessful for the reaction in water. Part of the catalyst remained in solution after centrifugation. However, an interesting phenomenon throwing light on the catalyst behavior during the reaction has been revealed. It was found, that the smaller quantity of the catalyst (~25%) remaining in solution after centrifugation appears to be much more active (approximately 2.5 times) than the precipitated catalyst (~75%). Moreover, during the subsequent recycling it has been found, that the precipitated catalyst has substantially lost its activity. TEM studies showed that the size of the PdNPs in the residue increases considerably after the reaction: 2.4–3.1 nm after the first cycle in comparison to 1.7 nm in the initial catalyst (Figure 5).

To understand which processes influence the change of the size and morphology of the catalyst, we have studied separately the action of all the reagents of the Suzuki-Miyaura reaction on the initial PdNPs. It was found, that addition of ArB(OH)_2_ and PhCOOH with KOH does not lead to any changes, but the addition of ArI (in our case ArI = *m*-IC_6_H_4_COOH) in the presence of KOH changes the nature of the catalyst. The formation of ultra small (0.6–0.8 nm), and larger than initial particles (up to 3 nm) was observed. At the end of the Suzuki-Miyaura reaction we observed only particles of the larger size, *i.e.,* fine particles, owing to their hyperactivity, were involved first in the reaction and dissolved. Thus, the dissolution of nanoparticles under the action of ArI led to the formation of not only bigger but also of smaller sized PdNPs.

**Figure 6:**
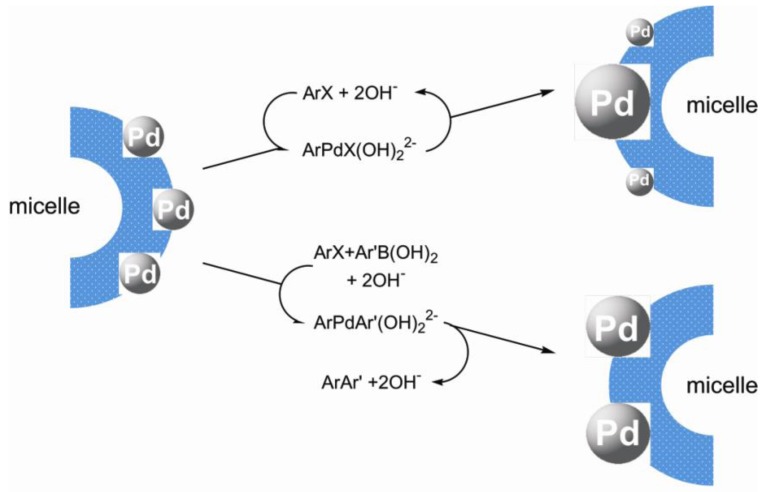
The prospective mechanism of Ostwald ripening of PdNPs at presence of ArX (at the left) and in the Suzuki-Miyaura reaction (at the right).

In our opinion, observable "grinding" of PdNPs occurred due to disproportionation of the PdNPs controlled by thermodynamics. Palladium transfer in this process was carried out with the participation of intermediates [ArPdX(OH)_2_]^2-^ and [ArPdAr’(OH)_2_]^2- ^in the catalytic cycles (Figure 6). Thus we come to conclusion, that an observable constancy of activity of the catalyst isolated by the ultrafiltration, is not related to preservation of the size and morphology of PdNPs, but is a result of crushing of these particles leading to more active smaller particles compensating for the lower activity of larger nanoparticles originated due to their growth and aggregation. Moreover, the analysis of this phenomenon leads us to a rather sad conclusion that it is impossible to expect an infinite number of recycles without a loss of catalytic activity due to increase in size of the nanoparticles caused by this process. Finally, large PdNPs turned out to be inactive or low active in the reactions [20].

**Figure 7:**
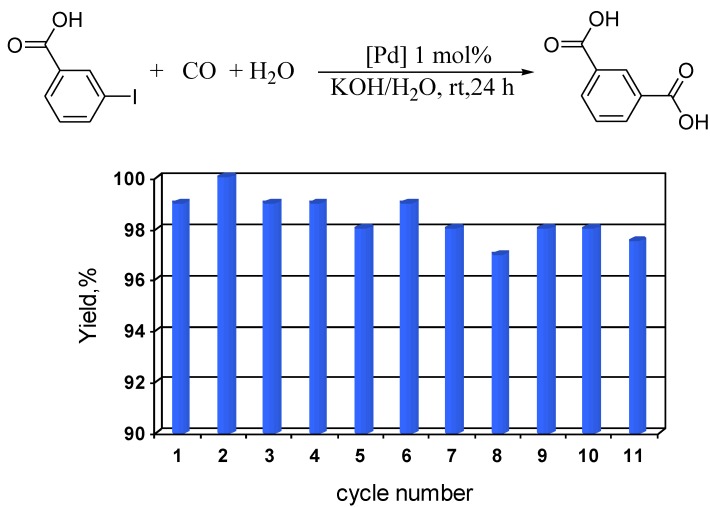
Hydroxycarbonylation of 3-iodobenzoic acid.

There are quite a few examples of the recycling of the catalyst in the carbonylation reaction despite its undoubtful importance [5,21,22]. One of them includes the above-mentioned microemulsion [13]. Use of the Pd-PS-PEO catalyst in hydroxycarbonylation reactions gave excellent results. The hydroxycarbonylation of *meta*- and *para*-iodobenzoic acids was carried out in water at room temperature using PdNPs micellar catalyst and KOH as a base [23]. Both reactions proceeded with full conversion to give quantitative yields of phthalic acids after 24 h. For 3-iodobenzoic acid, the catalyst was recycled 11 times, with full isolation of the product in the first five cycles and spectroscopic determination of yields in the final six cycles (Figure 7). 

### 2.3. Palladium nanoparticles immobilized on poly(N-vinylimidazole) polymer

The diblock copolymer micellar system described above can only be successfully recycled from reactions taking place in aqueous and alcoholic solutions and at temperatures below 60 ºC. Another soluble polymeric system based on poly(*N*-vinylimidazole) **(**PVI**)** proved to be much more stable and can be used under a broader range of conditions. We investigated PVI and its statistical copolymer with *N*-vinylcaprolactam poly(*N*-vinylimidazole-co-*N*-vinylcaprolactam) **(**PVI-PVC**)** (30 mol% PVC) as supports for PdNPs. 

**Scheme 4:**
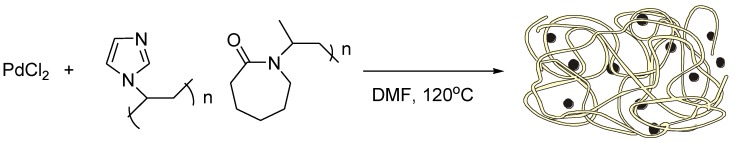
Scheme of the formation of the catalytic system Pd-(PVI-PVC).

**Figure 8:**
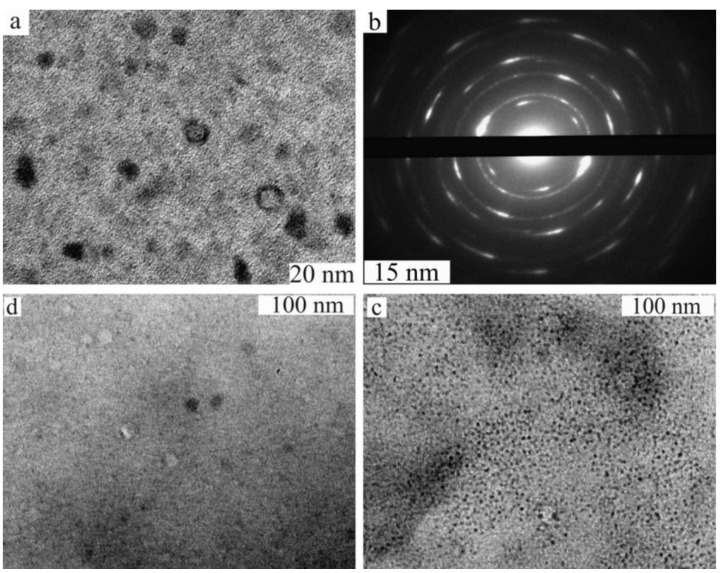
TEM images of the catalyst Pd-PVI-PVC (1:5): **(a) **after the reaction with PhI and *n-*butyl acrylate; **(b) **a diffraction pattern; **(c)** after heating in DMF for 1 h; **(d)** after the reaction of cyanation of *p*-bromoacetophenone.

Complexes of PVI and PVI-PVC with palladium dichloride were synthesized using the ligand donor ability of imidazole links. The subsequent research has shown that the complexes effectively catalyze the Heck reaction, during which the bivalent palladium is reduced to a zero-valent palladium forming PdNPs (Figure 8), stabilized by the polymer [24]. The same system is formed upon heating the complex of PdCl_2_ with polymer in DMF without other reagents (Scheme 4). Palladium(II) is probably reduced to palladium(0) nanoparticles with the admixture of dimethylamine present in or formed from DMF on heating [25]. The polymeric system with nanoparticles is easily separated from low-molecular products of reaction by precipitation with ether and as it has been shown, can be used several times without appreciable loss of activity. The system works stably both in aqueous and in organic (dimethylformamide) solutions, and practically in all the typical temperature range of palladium catalytic reactions: from room temperature up to 140 ºC. Catalytic properties of the PdNPs immobilized on *N*-vinylimidazole polymers have been investigated in a number of model reactions, including the Heck reaction, cyanation and carbonylation of aryl halides.

#### 2.3.1. The Catalysis of the Heck reaction

**Scheme 5:**
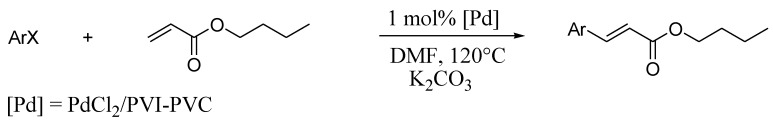
The Heckreaction.

In the course of Heck reaction between iodobenzene and *n-*butyl acrylate in dimethylformamide at 100–120 ºC in presence of the base (K_2_CO_3_), the complex of palladium dichloride with PVI is converted into PdNPs immobilized on PVI, which effectively catalyze the reaction [24]. High yields of the product of the arylation of alkene were observed for aryl iodides both with electron acceptor and electron donor substituents (Scheme 5).

Longer times and higher temperatures (140 ºC), which can be lowered to 120 ºC by addition of Bu_4_NBr, were required for aryl bromides. It has been shown for both reactions with PhI, and with *p*-AcC_6_H_4_Br, that recycling of the catalyst can be performed for at least five cycles without loss of activity. The separation of the catalyst was achieved by precipitation from a solution in DMF with ether. That fact, that after practically full precipitation of the catalyst, the negligible part remaining in the solution can catalyze the reaction rather effectively, serves as an indirect confirmation, that the process of particle “grinding”, similar to one observed by us for Suzuki reaction [20], is also important in this reaction.

#### 2.3.2. Cyanation of aryl halides

**Scheme 6:**
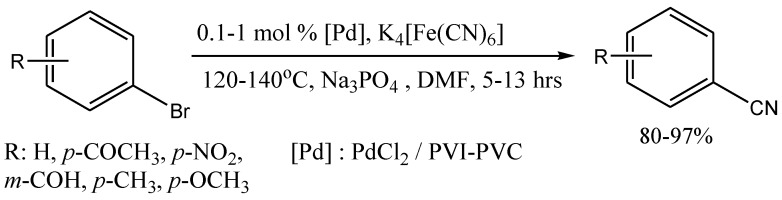
A cyanation reaction.

Cyanation of aryl halides is a useful reaction for the synthesis of drugs and dyes. A catalytic way of carrying out this reaction has become an important achievement and a lot of works devoted to catalyzed cyanation have been published. Recent advances include the use of the nontoxic source of cyanide ion K_4_Fe(CN)_6_ [26]. Cyanation with immobilized palladium (Pd/C) was reported, but the yield decreased after recycling the catalyst [27]. We used the system PdCl_2_-PVI-PVC with K_4_Fe(CN)_6_ as a source of cyanide ion in the catalytic cyanation of aryl bromides, aryl iodides and activated aryl chlorides [25] The reaction proceeds at 120-140 ºC to give high yield of aryl cyanides for both ArI and ArBr (Scheme 6).

It has been shown, that the catalyst can be easily separated from the reaction product, and can be used repeatedly. In the reaction with *p*-AcC_6_H_4_Br during nine consecutive cycles the yield remained in the 93–99% range.

#### 2.3.3. Methoxycarbonylation of aryl iodides

The efficient hydroxycarbonylation of aryl iodides catalyzed by Pd-PS-PEO-CPC prompted us to carry out alkoxycarbonylation in methanol using PdCl_2_-PVI-PVC as a pre-catalyst [23]. The study of reaction conditions has shown that the best result can be obtained with 1 mol% Pd/5 mol% PVI-PVC at 55 ºC and 1 atm CO using Et_3_N as a base (Table 3, entry 12). Recycling of the catalyst was done for several ArI. The yields were the same after three runs for neutral and electron deficient ArI substrates, but slowly decreased for electron rich ones (Table 4).

**Scheme 7:**
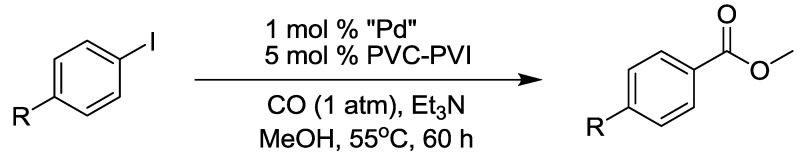
Methoxycarbonylation of aryl iodides.

**Table 3:** Methoxycarbonylation of aryl iodides catalyzed by polymer-supported palladium.

Entry	Base	Temp, ºC	Pressure of CO, atm	Yield, %
1	KOH	50	1	11
2	K_2_CO_3_	50	1	18
3	Et_3_N	50	1	32
4	KOH	50	1	45
5	K_2_CO_3_	50	1	53
6	Et_3_N	50	1	85
7	Et_3_N	50	1	70
8	Et_3_N	50	5	48
9	Et_3_N	50	30	41
10	Et_3_N	25	1	8
11	Et_3_N	40	1	63
**12**	**Et_3_N**	**55**	**1**	**92**

**Table 4:** Recycling the catalyst in the methoxycarbonylation reaction.

Entry	R	Yield of RC_6_H_4_C(O)OMe, %		
		Cycle 1	Cycle 2	Cycle 3
1	H	95	92	93
2	Me	92	92	90
3	OMe	91	89	83
4	C(O)Me	97	95	94

Thus, these novel Pd-PS-PEO-CPC and Pd-PVI-PVC catalytic systems with immobilized palladium allow us to perform reactions in water, methanol, and other organic solvents without requiring toxic phosphine ligands and to carry out the recyclization of the catalyst several times. 

## 3. Poly(*N*-vinylimidazole) in Organic Catalysis

The special properties of polymeric systems based on PVI are not limited to a role as the support for nanoparticles. It is known that imidazole is a fragment of the side chain of the amino acid residue of histidine which is a part of practically all enzymes, and is capable of catalyzing a number of biochemical processes [28]. Besides, a PVI-based polymer successfully models natural enzymes on the basis of peptide macromolecules [29,30]. The behavior of the amphiphilic copolymeric PVI-PVC system is similar to that of polypeptides, and regulation of the ratio of imidazole and caprolactam monomeric links allows one to model different types of polypeptides differing in their amino acid composition and their hydrophobicity. Considering the opportunities of catalysis of certain types of reactions by imidazole, we investigated the catalysis by PVI in similar transformations. Advantages of PVI are its solubility in water and aqueous solutions and possibility to be recycled.

In aqueous solutions the polymers form aggregates where hydrophobic reagents are concentrated which leads to an increase of the rate of reactions between them by several orders of magnitude. The interfacial nanolayer of aggregates can be considered as a catalytic nanoreactor. PVI and PVI-PVC polymers were studied as catalysts for the hydrolysis of esters [30]. We investigated the application of the polymer in organic catalysis, particularly in the reactions of nucleophilic addition to electron deficient olefins. Nucleophiles based on SH, NH and CH acids were deprotonated with PVI acting as polymeric base, followed by their attack on the C=C double bond. Protonation of the intermediate gave the product. The reactions proceeded in water or water-ethanol mixtures at room temperature providing high yields of the products. The catalyst was easily separated from the reaction and can be reused many times.

We used PVI with molecular weight 75,300 as the recycled organic catalyst essentially accelerating the addition of thiols to Michael acceptors in aqueous or aqueous ethanol solutions at room temperature [31]. Michael addition of thiophenol under the described conditions proceeded smoothly with a number of electron deficient olefins. Not only the thiophenol, but also alkylthiols reacted under these conditions. As it has been already noted, the catalyst was easily separated from products and can be used multiple times.

**Scheme 9:**
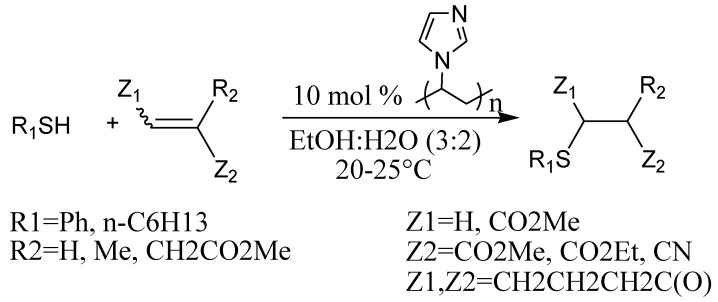
Michael addition of thiophenol to electron deficient olefines.

**Scheme 10:**
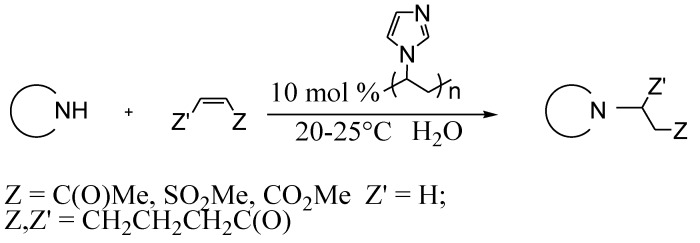
Michael addition of NH-acids to electron deficient olefins.

**Scheme 11:**
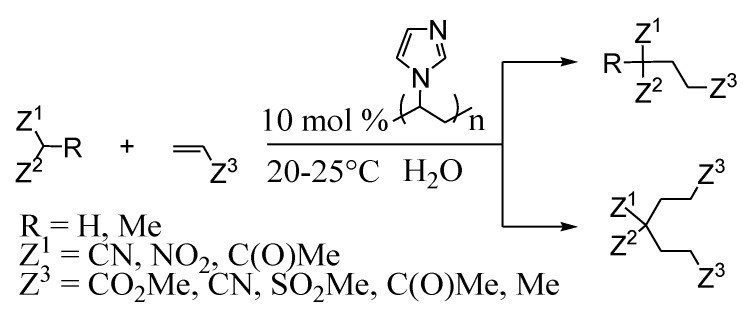
Michael addition of CH-acids to electron deficient olefins.

Michael addition of a number of NH-acids (azoles) [32] (Scheme 10) and CH-acids [33] (Scheme 11) proceeded analogously to give corresponding products with high yields.

Thus, it has been shown that PVI can be used as inexpensive, effective, and moreover recyclable organic catalyst in reactions of addition of aromatic and aliphatic nucleophiles based on SH-, NH- and CH-acids to Michael acceptors, allowing one to carry out reactions in water or aqueous mixtures.

## 4. Bimetallic Nanoparticles

**Scheme 12:**
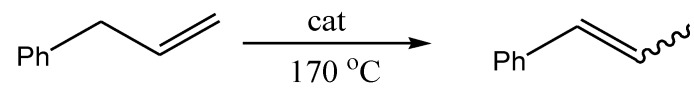
The reaction of isomerization of allylbenzene to β-methylstyrene.

Bimetallic nanoparticles were recently discovered to exhibit superior catalytic activity and improved deactivation resistance compared to monometallic nanoparticles in many systems when they were used as catalysts. Improved activity of bimetallic nanoparticles as well as selectivity towards desired products can be result of synergetic electronic interactions between two different metal atoms in the individual nanoparticles. The catalytic properties of bimetallic nanoparticles can often be tuned by changing the composition of the bimetallides [34]. The catalytic activity of nanosized particles of Au, Ni, Co, Fe, Pd and their bimetallides Au-Ni, Au-Fe, Au-Co, Au-Pd, immobilized on a support (γ-Al_2_O_3_, SiO_2_) was compared with each other in the reaction of isomerization of allylbenzene to β-methylstyrene (Scheme 12) [35].

Metal vapor synthesis was used for obtaining the bimetallides of gold [36]. The composition and structure of nanocomposites were investigated by methods of the nuclear absorption, TEM, X-ray photoelectron spectroscopy, and X-ray phase analysis. From the listed metals only Pd and Au showed certain catalytic activity. Activity of such bimetallides as Au-Co, Au-Fe was determined exclusively by the Au contents in them, and activity of the Au-Pd bimetallide was the sum of activity of the separate metals. However, for Au-Ni system the activity considerably exceeded the activity of Au and Ni, i.e. a strongly pronounced synergetic effect was observed. At the same concentration activity of Au-Ni appeared higher than activity of Pd by 50-fold, Au-Pd - by 100-fold, Au - by 500-fold. [35].

## 5. Nanostructured Metal Salts

**Scheme 13:**
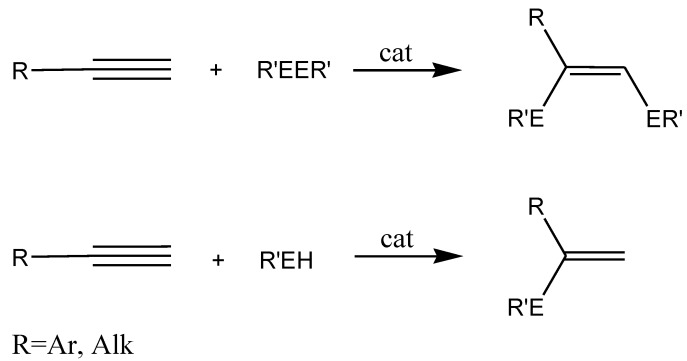
The addition reactions of E-E and E-H bonds to alkynes.

The synthesis of vinyl derivatives of various elements (formation of С_sp2_-heteroatom bonds) can be performed not only as a result of catalytic cross-coupling reaction, but also by addition reactions of E‑E or E-H bonds to alkynes (E-heteroatom). Such a process is preferable from the Green Chemistry point of view in comparison to substitution reactions as it proceeds with 100% atom efficiency. Use of transition metals complexes as catalysts allows one to carry this out, unlike radical reactions, in a regio- and stereoselective manner. Thus, in studying such reactions with participation of Ar_2_E_2_ or ArEH (E = S, Se) we found conditions for obtaining exclusively the *syn*-products of addition in the first case and exclusively Markovnikov products (α-isomer) - in the second (Scheme 13).

**Figure 9:**
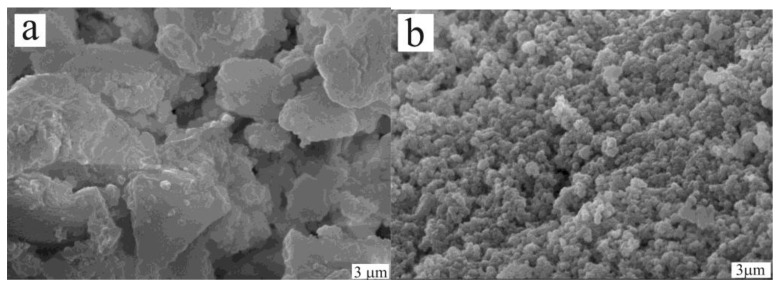
SEM images of the catalyst obtained from **(a)** Pd(OAc)_2_**, (b) **Ni(acac)_2_.

It turned out that the first reaction can be performed in the molten state with small amount of PPh_3_ without solvent using homogeneous complexes of palladium with phosphine ligands [37,38]. Correct selection of ligands allowed alkyl derivatives AlkEEAlk to participate in the reaction for the first time [39]. However, in the second reaction the presence of a ligand led to a side process of formation of a bis(sulphur or selenium)-substituted alkene. At the same time without a ligand there was formation of insoluble polymer [Pd(SAr)_2_]_n_ which was not active in the reaction of addition of Ar_2_E_2_ and weakly active in the reaction of addition of ArEH. The problem has been solved by use of the heterogeneous catalyst [Ni(SAr)_2_]_n_ made from Ni(acac)_2_ and ArSH at presence of an alkyne [40,41]. SEM studies showed that only in this case well formed particles close in size to nanostructures were formed (Figure 9b). 

**Scheme 14:**
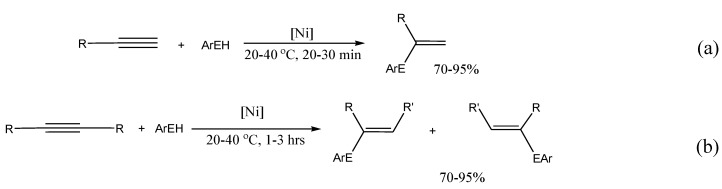
The addition reactions catalyzed by the nanostructured Ni catalyst.

Investigation of this catalyst revealed that its activity so exceeded the activity of its homogeneous analogue with carbene ligands CpNi(NHC)Cl (NHC = *N*-heterocyclic carbene) [42], that reactions can go at room temperature. Moreover, with participation of this catalyst for the first time it became possible to carry out addition reactions of ArEH not only to terminal alkynes, but also to internal alkynes (Scheme 14) [43]. 

**Figure 10:**
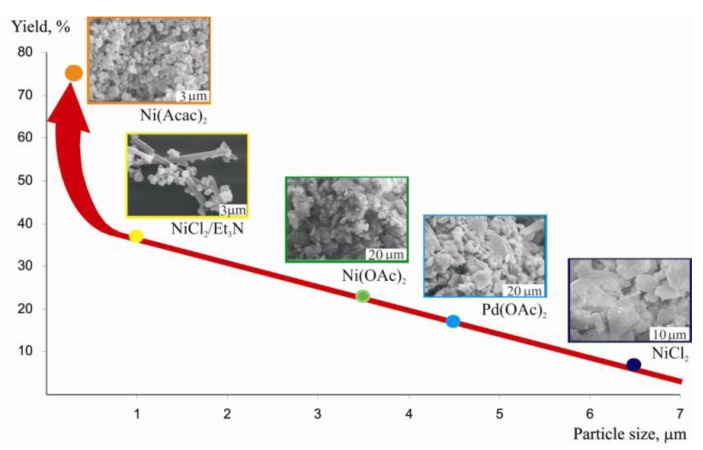
Dependence of yield of the reaction on the size and morphology of catalytic particles [M(SAr)_2_]*_n_* derived from different precursors MX_2_.

That fact, that from all precursors of nickel complex NiX_2_, complexes of identical composition [Ni(SAr)_2_]*_n_*, but of different activity were formed, has been explained only on the basis of study of the structure of particles. It has been shown, that the catalytic activity directly depends on the sizes of particles and their morphology. The curve showing the dependence of the yield from the size of particles has both linear and exponential regions (Figure 10). 

**Figure 11:**
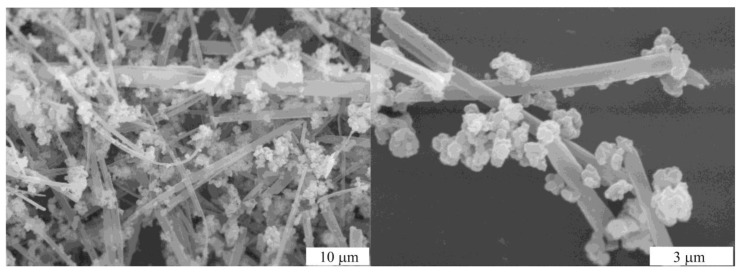
SEM images of the catalyst obtained from NiCl_2_/Et_3_N.

Only from Ni(acac)_2_ were well organized particles with the highest activity obtained. Addition of Et_3_N to NiCl_2_ resulted in formation of Et_3_N∗HCl [44] forming “branches” preventing Ni nanoparticles from aggregation and that also helped to increase catalytic activity (Figure 11).

**Figure 12:**
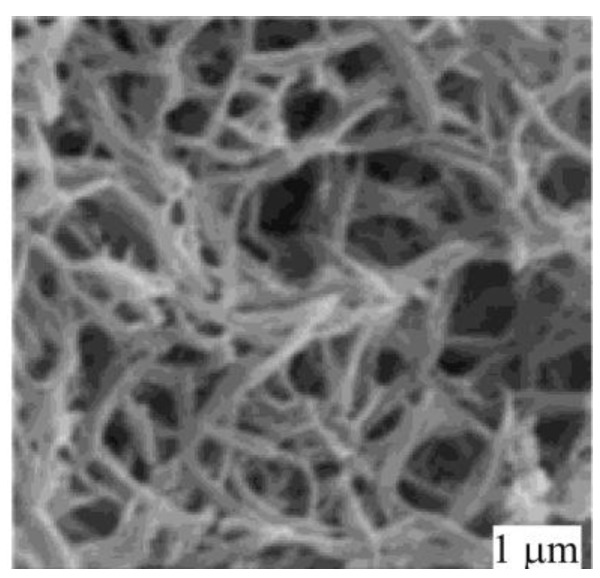
Microphotograph of [Pd(SCy)_2_]*_n_* obtained from Pd(OAc)_2_

We have solved the problem of regioselective addition of aliphatic derivatives AlkEH to terminal alkynes using [Pd(SAlk)_2_]*_n_* obtained from Pd(OAc)_2_ as a catalyst. The most active catalysts [M(SAlk)_2_]*_n_* in case of SАlk ligands turned out to be not complexes of nickel, but rather palladium complexes [45]. As one can see in the image obtained by means of SEM, [Pd(SCy)_2_]*_n_* synthesized from palladium acetate has perfectly formed fine structured wires (Figure 12). It is interesting to note that only palladium salts demonstrated perfect catalysis, allowing one to obtain alkyl vinyl sulfides in high yields from various terminal alkynes. At the same time Ni salts produced “ugly” particles with low catalytic activity in this reaction. Thus, two important synthetic problems have been solved, and these reactions have been shown to be easily scaled.

**Scheme 15:**
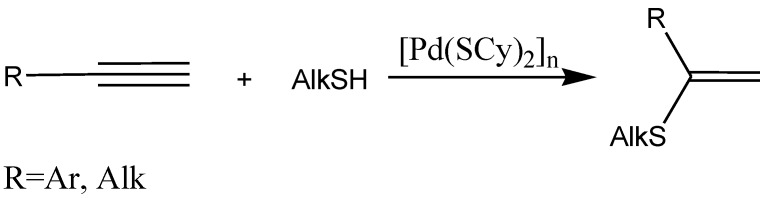
The addition of aliphatic derivatives AlkEH to terminal alkynes.

As in the case of metal nanoparticles considered in the previous sections, for the particles consisting of metal salts, one of the most important questions is that of the nature of the active form of the catalyst. The techniques of hot filtration and hot centrifugation of the catalytic mixtures gave evidence that the reactions catalyzed by nanostructured metal salts proceed on the surface of the catalyst and leaching does not ocurr in the reactions [40,41]. Addition of ligands like PPh_3_ can destroy the coordination of the salt to the polymeric chain leading to its partial dissolution, but in this case the concurrent reaction of formation of disubstituted olefins takes place.

## 5. Conclusions

This work clearly demonstrates that on the basis of nanoparticles of different type one can obtain effective catalysts (metal or organic) of fine chemicals production. It also shows that catalytic activity depends not only on the nature of the metal, but also on the size and morphology of the particles. The possibility of recycling the catalyst is determined by the nature of the process: does it take place on the surface of the particles or in the bulk solution. In the latter case leaching and particle growth limit the recycling. Organocatalysts can be easily recovered and reused. As to metal nanoparticles, they can also be recycled, but due to leaching and the homogeneous nature of our reactions the number of cycles is limited.
